# Effects of Reactive MgO on the Reaction Process of Geopolymer

**DOI:** 10.3390/ma12030526

**Published:** 2019-02-10

**Authors:** Zhaoheng Li, Wei Zhang, Ruilan Wang, Fangzhu Chen, Xichun Jia, Peitong Cong

**Affiliations:** 1 College of Water Conservancy and Civil Engineering, South China Agricultural University, Guangzhou 510642, China; lizhaoheng2008@163.com (Z.L.); zhangwei1@yahoo.com (W.Z.); chenfangzhu@163.com (F.C.); jiaxichun@stu.scau.edu.cn (X.J.); 2 Guangdong Research Institute of Water Resources and Hydropower, Guangzhou 510635, China; wangruilan@163.com

**Keywords:** reactive MgO, geopolymer, shrinkage, reaction process

## Abstract

In order to compensate for the shrinkage of geopolymer pastes uniformly, reactive MgO powders are evenly dispersed in the geopolymer. The deformation performance, mechanical properties, microstructure and components of geopolymer pastes with reactive MgO are characterized. The effects of the content and the activity of MgO are discussed. The results indicate that the chemical shrinkage, autogenous shrinkage and drying shrinkage decrease with the addition of reactive MgO. MgO reacted with water, and fine Mg(OH)_2_ crystals forms as a geopolymer paste. Mg(OH)_2_ produces uniform expansion, which refines the pore size of pastes and the compressive strength increases. The shrinkage of the geopolymer pastes is thus effectively compensated.

## 1. Introduction

Geopolymer is mainly composed of industrial by-products such as fly ash, slag, metakaolin and alkaline activators. Compared to Portland cement, geopolymer has lower energy consumption and CO_2_ emissions. In addition, it presents high early strength, a compact structure and better corrosion resistance. Therefore, geopolymer has attracted wide attention worldwide and shows broad application prospects and potential [1,2,3,4]. However, it has also been reported [5,6] that geopolymers have some potential problems such as efflorescence, large drying shrinkage, carbonization and potential alkali leaching. Most of these problems can be effectively improved by taking some measures, for example, by adding silica fume and adjusting the activator content, the amount of activator required to inhibit efflorescence can be effectively inhibited [7]. In addition, carbonation can be alleviated by increasing the strength and improving the microstructure of geopolymer concrete [7,8].

Recent research has shown that [9,10,11,12] the drying shrinkage of geopolymer paste was much larger than that of Portland cement paste, which was 500 × 10^−6^–2000 × 10^−6^ at 7 days and 2800 × 10^−6^ at 28 days, about five times that of the drying shrinkage in Portland cement [13]. Further, geopolymer exposure to a lower relative humidity resulted in even higher drying shrinkage than the Portland cement. The difference between them will further expand with the decrease of relative humidity [14,15,16]. The difference in drying shrinkage between the geopolymer paste and the Portland cement paste is mainly caused by the pore structure and gel content [17,18,19]. The porosity of geopolymer paste is usually lower than that of Portland cement paste. The pores are mainly small pores with a pore diameter of about 10 nm. Geopolymer paste contains more gel than Portland cement, so the shrinkage deformation is larger [20,21]. Geopolymer is quick in curing and hardening, large in shrinkage and easy to crack, and thus elucidating how to improve the volume stability of geopolymer is very important to prevent cracking of geopolymer concrete and improve its durability [22,23,24].

At present, the volume deformation of geopolymer is mainly controlled by reducing the content of soluble silicate and alkali in the activator, but this improvement is limited. Among the measures to improve the volume stability of cementitious materials, adding a shrinkage compensation component is a common method. The components of compensating shrinkage can be divided into three categories: Calcium oxide, calcium sulphoaluminate and MgO [25]. Calcium oxide and calcium sulphoaluminate react violently and are not easily controlled in a high alkali environment, thus they cannot achieve a good compensating shrinkage effect in geopolymer [26,27,28,29]. 

In this study, the deformation performance, mechanical properties, microstructure and components of geopolymer pastes with reactive MgO are characterized, and the effects of the content and the activity of MgO are discussed. By controlling the activity and particle size of MgO, the shrinkage compensation effect of MgO is evaluated.

## 2. Materials and Methods

### 2.1. Raw Materials

**Figure 1:**
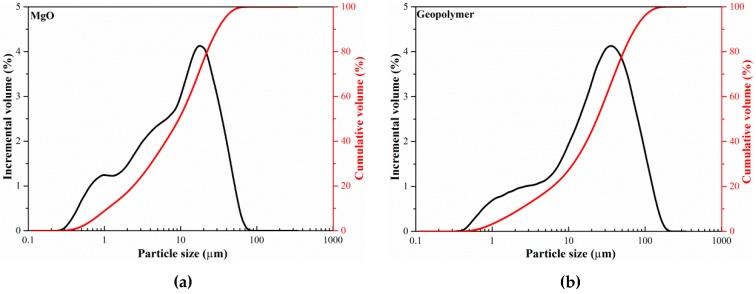
Particle size distribution of reactive MgO (**a**) and geopolymer (**b**).

The reactive MgO (with activities of 60 s, 150 s and 220 s) used in this study was a product from the Wuhan Sanyuan Special Building Materials Co., Ltd. (Wuhan, China). The geopolymer is composed of slag, fly ash, silica fume, and the activator and reinforcing components, with a mass ratio of 70:12:5:8:5. The activator is composed of sodium silicate and sodium carbonate. The chemical composition and physical and mechanical properties of the geopolymer are shown in Table 1 and Table 2. The particle size distribution of reactive MgO and the geopolymer are shown in Figure 1. As can be seen from Figure 1, the particle sizes of geopolymer particles (10–40 μm) and the particle sizes of reactive MgO (mainly distributed in 5–20 μm) are relatively small.

### 2.2. Testing Methods

The geopolymer was mixed with 4–12% (mass fraction, based on the total mass of geopolymer and reactive MgO) of reactive MgO with different activities (shown in Table 3) and stirred evenly, and geopolymer without reactive MgO was define as Ref., according to the water/solid ratio of 0.38 (when the fluidity of mortar jumped to 180 ± 5 mm), the compressive strength and flexural strength were measured according to American Society for Testing Material (ASTM) method C 349 at each curing age (days 3, 7 and 28), and the drying shrinkage was tested with reference to JC/T603-2004 "Test Method for Dry Shrinkage of Cement Mortar" [30]. The matching of geopolymer paste for the test is shown in Table 3.

When testing autogenous shrinkage, the paste was sealed in the bellows, the volume deformation was converted from the bellows to the length deformation, the length deformation was converted into a voltage signal by a displacement sensor, then converted into a digital signal by an analog-to-digital converter and then transmitted to a computer. Finally, the data were recorded in real-time with software. The chemical shrinkage test was carried out according to ASTM C 1608-07. The geopolymer and water (with a water/solid ratio of 0.38) were first mixed into a paste and then carefully added into 500 mL glass bottles. After the glass bottle was filled with water, the glass bottle was sealed with a rubber stopper with a glass scale tube (maximum capacity of 5 mL, accuracy of 0.01 mL) and a few drops of paraffin oil were dropped into the glass scale dropper to prevent water evaporation during the experiment. 

After curing for a certain period of time, the paste was crushed, the intermediate sample was sprayed with carbon or gold and the morphology of the hydrated product was observed by the EVO MS18 scanning electron microscope (SEM) of Zeiss (Jena, Germany). The element composition in the microarea of the sample was determined by a X-Max energy spectrum analyzer from the Oxford Company (Oxford, UK). The mineral composition of geopolymer paste was characterized by a Xpert Pro X-ray diffractometer (XRD) from PANLYTICAL (Almelo, The Netherlands). The thermal analysis curve of the geopolymer paste was determined by the STA 449 C thermal analyzer (DSC/TG) from the Netzsch Company (Selb, Germany) and the content of Mg(OH)_2_ was characterized by weight loss at 300–400 °C. The porosity of hardened paste was determined by using an AutoPore IV 9500 mercury porosimeter from the Micromerics Company (Norcross, GA, USA). For the test parameters, the pore size range was 0.003–1000 μm, the maximum pressure was 228 MPa and the volume accuracy of mercury injection or mercury withdrawal was greater than 0.1 µL.

## 3. Results and Discussion

### 3.1. Effect of MgO on the Deformation of Geopolymer Paste

#### 3.1.1. Effect of MgO on the Chemical Shrinkage of Geopolymer Paste

**Figure 2:**
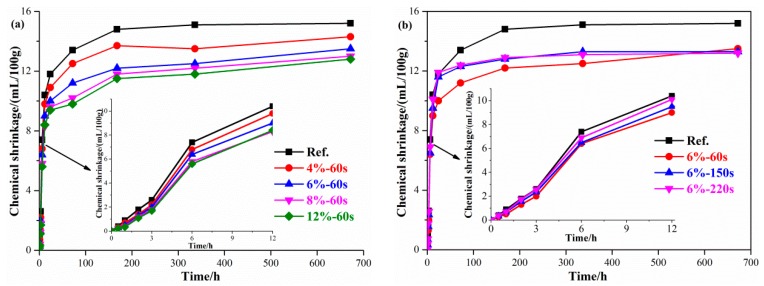
Effects of the reactive MgO on the chemical shrinkage of geopolymer. (**a**) Content of MgO. (**b**) Reactivity of MgO.

The chemical shrinkage of geopolymer paste refers to the reduction of the absolute volume of the reaction product by the sum of the absolute volumes of cement and water before hydration. The chemical shrinkage of geopolymer paste mixed with reactive MgO is shown in Figure 2. The chemical shrinkage of the geopolymer pastes increased rapidly after mixing with water, with a chemical shrinkage value of 15 mL/100 g after 7 days, after which it tended to increase steadily. As shown in Figure 2a, adding reactive MgO to a geopolymer could improve the chemical shrinkage of the geopolymer paste. The chemical shrinkage decreases gradually with the increase of reactive MgO content. The 7 day chemical shrinkage of geopolymer paste mixed with 6% and 12% reactive MgO decreased by 17.6% and 20.9%, respectively, and the 28-day chemical shrinkage decreased by 10.7% and 14.7%, respectively. As shown in Figure 2b, MgO with high-activity (60 s) promoted the compensation for early chemical shrinkage of the geopolymer paste, while MgO with low-activity (150 s and 220 s) reduced the chemical shrinkage of the geopolymer paste after 28 days.

#### 3.1.2. Effect of MgO on the Autogenous Shrinkage of Geopolymer Paste

**Figure 3:**
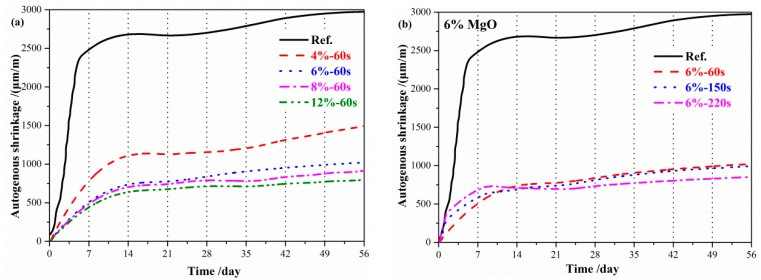
Effects of the reactive MgO on the autogenous shrinkage of the geopolymer: (**a**) Content of MgO; (**b**) reactivity of MgO.

The autogenous shrinkage of geopolymer paste refers to the macroscopic volume of geopolymer paste that decreases due to the reaction after initial setting. The autogenous shrinkage of geopolymer paste mixed with reactive MgO is shown in Figure 3. The autogenous shrinkage of geopolymer paste was larger, reaching 1500 μm/m after 1 day, and still increasing significantly after 7 days. As can be seen from Figure 3a, the autogenous shrinkage gradually decreased and tended to be gentle after 24 h with the increase of reactive MgO content. The autogenous shrinkage of geopolymer paste cured for 3 days and 7 days decreased by 64.6% and 72.1%, respectively, when the content of reactive MgO was 6%. The autogenous shrinkage of geopolymer paste cured for 3 days and 7 days reduced by 71.4% and 78.3%, respectively, when the content of reactive MgO was 12%, indicating that reactive MgO can effectively compensate the autogenous shrinkage of geopolymer paste. Figure 3b indicates that MgO with high-activity (60 s) reduced the early autogenous shrinkage (0–14 days) of the geopolymer paste, while MgO with low-activity (150 s and 220 s) decreased the autogenous shrinkage of the geopolymer paste after 14 days.

#### 3.1.3. Effect of MgO on the Drying Shrinkage of Geopolymer Paste

**Figure 4:**
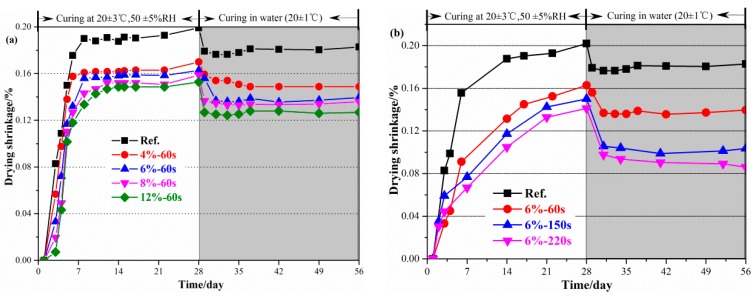
Effects of the reactive MgO on the drying shrinkage of geopolymer: (**a**) Content of MgO; (**b**) reactivity of MgO.

The drying shrinkage of geopolymer paste refers to the contraction caused by the evaporation of internal moisture, which occurs when the external environment humidity is lower than the internal humidity of the geopolymer paste. The drying shrinkage of geopolymer paste mixed with reactive MgO is shown in Figure 4. The drying shrinkage of geopolymer paste increased rapidly with an increase of curing time and the maximum drying shrinkage reached 0.19%. The drying shrinkage of the hardened paste decreased significantly with an increase of reactive MgO content. The drying shrinkage of hardened paste decreased by 20.0% after 28 days, when the content was 6%. As shown in Figure 4b, MgO with high-activity (60 s) reduced the early drying shrinkage (0–7 days) of the geopolymer paste, while MgO with low-activity (150 s and 220 s) decreased the drying shrinkage of the geopolymer paste after 7 days. The sample was placed in water and the irreversible shrinkage of geopolymer paste was determined after 28 days. The irreversible shrinkage of geopolymer paste was much higher than that of the geopolymer paste with reactive MgO, indicating that reactive MgO can better compensate for the drying shrinkage in an environment with sufficient moisture.

### 3.2. Effect of MgO on the Mechanical Properties of Geopolymer

**Figure 5:**
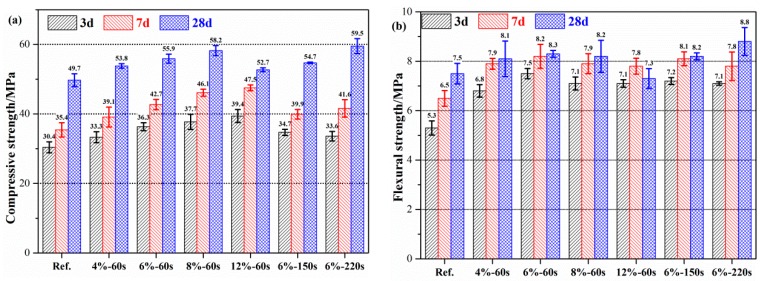
Effects of the reactive MgO on the mechanical properties of geopolymer: (**a**) Compressive strength; (**b**) flexural strength.

The mortar strength of geopolymer mortar with MgO is shown in Figure 5. The early compressive strength and flexural strength of geopolymer mortar (before 7 days) increased with an increase in the content of reactive MgO, however the compressive strength and flexural strength after 28 days increased first and then decreased. The compressive strength of the geopolymer after 3 days, 7 days and 28 days increased by 12.4%, 8.1% and 12.8%, respectively, however, when the content of reactive MgO was 8%, the flexural strength was increased by 18.6%, 14.5% and 1.3%, respectively. Although, after day 28, the compressive strength and flexural strength were reversed when the content of reactive MgO was 12%, indicating that an excessive content of reactive MgO is not conducive to the strength development of geopolymer mortar, and the appropriate content is 4%–8%. Mg(OH)_2_ was generated when the content of reactive MgO was 12%. Excess Mg(OH)_2_ was not conducive to the mechanical properties. Figure 5b indicated that MgO with high-activity (60 s) increased the early mechanical properties (3 days) of the geopolymer mortar, while MgO with low-activity (150 s and 220 s) improved the mechanical properties of the geopolymer mortar after 28 days.

### 3.3. Composition and Structure of Geopolymer Paste

**Figure 6:**
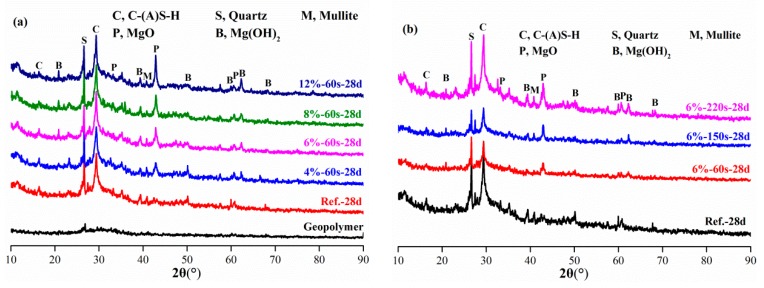
XRD patterns of geopolymer pastes with reactive MgO. (**a**) Content of MgO. (**b**) Reactivity of MgO.

The XRD analysis results of geopolymer paste with reactive MgO are shown in Figure 6. Geopolymer paste cured for 28 days (Ref.-28d) mainly contains C-S-H gel, C-A-S-H gel, Mg(OH)_2_, MgO, mullite and quartz. The XRD analysis results of geopolymer pastes with different reactive MgO contents are shown in Figure 6b. The formation of Mg(OH)_2_ also occurred with different reactive MgO additions.

**Figure 7:**
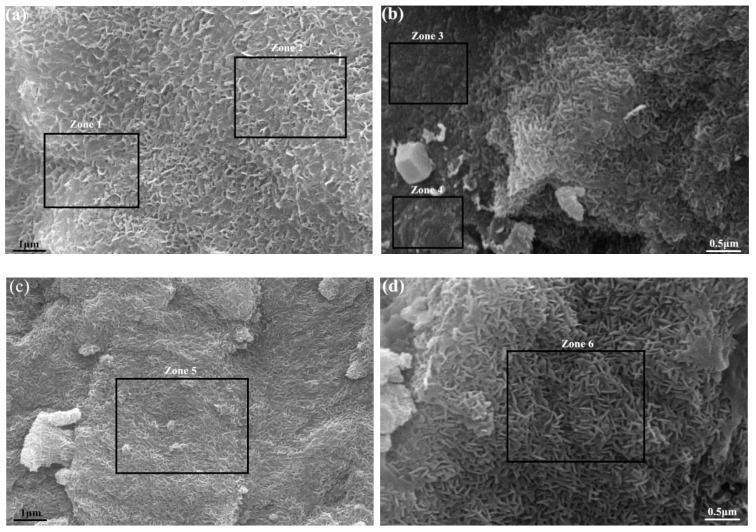
SEM images of hardened geopolymer pastes with reactive MgO cured for 28 days: (**a**) Ref.-28 days; (**b**) 6%-60 s-28 days; (**c**) 6%-150 s-28 days; (**d**) 6%-220 s-28 days.

The SEM images of geopolymer hardened pastes with different MgO contents are shown in Figure 7. The hydration products of geopolymers were mainly C-(A)S-H gels [31]. Figure 7b shows that worm-like Mg(OH)_2_ appeared locally in the geopolymer paste. Figure 7c,d shows a large area of worm-like Mg(OH)_2_ that appeared in the geopolymer paste. By SEM-Energy Dispersive Spectrometer (EDS)(see the black boxes in Figure 7), the results show that the Mg content of the C-(A)S-H gel with a low Ca/Si ratio in the geopolymer paste was little or not detected, as shown in Table 4. The Mg content in the worm-like area in the geopolymer paste exceeds 40%, indicating that a large amount of worm-like Mg(OH)_2_ was generated.

**Figure 8:**
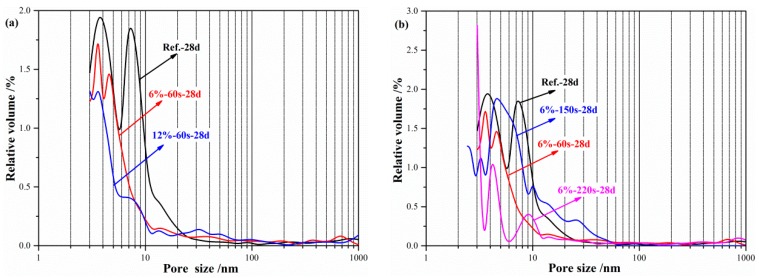
Effects of the reactive MgO on the pore size distribution of geopolymer: (**a**) Content of MgO; (**b**) reactivity of MgO.

The pore size distribution of the geopolymer paste with reactive MgO is shown in Figure 8. The pore size of the geopolymer paste cured for 28 days (Ref.-28d) was basically less than 30 nm. The pore size of the geopolymer paste was significantly refined with an increase of the reactive MgO content, and most of the pores were below 7 nm. These pore sizes indicate that reactive MgO can significantly improve the early and late densification of the geopolymer paste.

## 4. Conclusions

When the geopolymer was mixed with MgO and water, MgO hydrated to form brucite, which compensated the volume shrinkage of geopolymer pastes. The formation of brucite was confirmed via XRD and microstructure analyses, and the effect of the MgO on the reaction process of the geopolymer paste was discussed. XRD revealed the components of the reaction products, and SEM was found to be a useful tool for characterizing the morphology of the reaction products. The following conclusions can be drawn from the present study:

(1) The geopolymer solidified quickly and generated a large number of C-S-H and C-A-S-H gels, which show large chemical shrinkage, autogenous shrinkage and drying shrinkage at the early stage.
(2) Adding reactive MgO with fine particles into the geopolymer paste to generate a large number of fine worm-like Mg(OH)_2_ can result in uniform microexpansion, refine the pore size of pastes, increase the compressive strength, and effectively compensate the chemical shrinkage, autogenous shrinkage and drying shrinkage.
(3) With an increase of MgO content, the autogenous shrinkage, chemical shrinkage and drying shrinkage of geopolymer paste decreases gradually. The shrinkage of hardened geopolymer paste at early stage is small when adding high-activity MgO. The volume shrinkage of hardened paste at a later stage can be compensated by adding low-activity MgO.
(4) In the high alkalinity liquid phase environment of geopolymer, the generated Mg(OH)_2_ crystal is small and does not grow easily, which disperses in the geopolymer paste, resulting in uniform volume expansion, effectively compensating for volume shrinkage in the hardening process and matching the shrinkage process.

## Figures and Tables

**Table 1:** Chemical composition of geopolymer and reactive MgO (%).

Composition	SiO_2_	Al_2_O_3_	Fe_2_O_3_	TiO_2_	CaO	MgO	SO_3_	P_2_O_5_	K_2_O	Na_2_O	LOI
Geopolymer	30.56	19.56	2.22	0.85	34.40	3.23	1.23	0.05	2.09	4.88	0.93
MgO	2.02	1.24	0.94	-	2.40	92.26	-	-	-	-	1.14

NOTE: Loss on ignition (LOI).

**Table 2:** Physical and mechanical properties of geopolymer.

Standard Consistency Water Consumption	Specific Surface Area (m^2^/kg)	Density (g/cm^3^)	Soundness	Compressive Strength (MPa)			Flexural Strength (MPa)		
				3 d	7 d	28 d	3 d	7 d	28 d
0.224	370	2.91	Qualified	29.6	40.2	49.8	7.2	7.5	7.8

**Table 3:** Proportions of geopolymer pastes.

Number	Mass fraction of Geopolymer/%	Mass Fraction of Reactive MgO/%	Reactivity of MgO/s	Water-Solid Ratio
Ref.	100	0	/	0.38
4%-60s	96	4	60	0.38
6%-60s	94	6	60	0.38
8%-60s	92	8	60	0.38
12%-60s	88	12	60	0.38
6%-150s	94	6	150	0.38
6%-220s	94	6	220	0.38

**Table 4:** Element compositions of zones in SEM images of the geopolymer pastes.

Chemical Element	O	Na	Si	Ca	Al	Mg
Zone 1	69.91	3.66	8.46	11.30	4.69	1.98
Zone 2	70.28	4.74	7.38	10.29	5.21	2.10
Zone 3	69.71	3.86	8.16	11.60	4.99	1.68
Zone 4	70.08	4.94	7.28	10.39	5.11	2.20
Zone 5	42.10	3.30	6.47	2.51	3.64	41.98
Zone 6	46.28	2.69	5.19	2.21	2.82	40.15
