# Erratum: Li, Z.; Zhang, W.; Wang, R.; Chen, F.; Jia, X.; Cong, P. Effects of Reactive MgO on the Reaction Process of Geopolymer. *Materials* 2019, *12*, 526

**DOI:** 10.3390/ma12091388

**Published:** 2019-04-29

**Authors:** Zhaoheng Li, Kexin Zhao, Yongmin Yang

**Affiliations:** 1 Guangdong Research Institute of Water Resources and Hydropower, Guangzhou 510635, China; lizhaoheng2008@163.com; 2 South China University of Technology, Guangzhou 510640, China; mekxzhao@scut.edu.cn

**Author Contributions:** Conceptualization, Z.L.; Methodology, K.Z. and Z.L.; Software, Z.L.; Validation, K.Z.; Formal analysis, Z.L.; Investigation, Z.L.; Resources, Z.L.; Writing—Original draft preparation, Z.L.; Writing—review and editing, Y.Y.; Visualization, Y.Y.; Supervision, K.Z.; Project administration, Y.Y.; Funding acquisition, Y.Y.

**Funding:** This work was supported by the Natural Science Foundation of China, grant numbers 51802099 and 51172075, the Natural Science Foundation of Guangdong Province China, grant numbers 2018A030310389, and Water Resource Science and Technology Innovation Program of Guangdong Province, grant numbers 2015-10.

**Conflicts of Interest:** The authors declare no conflict of interest.

The authors wish to revise the authorship and the affiliations, due to the fact that we did not get the permission of the project leader of the Guangdong Water Conservancy Science and Technology Innovation Project (2015-10) in this published paper [1]. The previous authorship and affiliations were shown below:

Zhaoheng Li ^1,2^, Wei Zhang ^1^, Ruilan Wang ^2^, Fangzhu Chen ^1^, Xichun Jia ^1^ and Peitong Cong ^1,^*

^1^  College of Water Conservancy and Civil Engineering, South China Agricultural University, Guangzhou 510642, China; lizhaoheng2008@163.com (Z.L.); zhangwei1@yahoo.com (W.Z.); chenfangzhu@163.com (F.C.); jiaxichun@stu.scau.edu.cn (X.J.)
^2^  Guangdong Research Institute of Water Resources and Hydropower, Guangzhou 510635, China; wangruilan@163.com

* Correspondence: congpeitong@163.com

Received: 30 December 2018; Accepted: 1 February 2019; Published: 10 February 2019

The authors would like to apologize for any inconvenience caused to the readers by these changes.
